# Spectral domain optical coherence tomography features of multiple subfoveal retinal pigment epithelial tears after intravitreal bevacizumab

**DOI:** 10.4103/0301-4738.73712

**Published:** 2011

**Authors:** Kakarla V Chalam, Ravi K Murthy, Shailesh K Gupta, Vikram S Brar

**Affiliations:** Department of Ophthalmology, University of Florida-College of Medicine, Jacksonville, Florida

**Keywords:** Bevacizumab, retinal pigment epithelial tear, spectral domain optical coherence tomography

## Abstract

Retinal pigment epithelial (RPE) tear has been described to occur spontaneously, after laser photocoagulation and in recent times, after intravitreal injection of anti-vascular endothelial growth factor (VEGF) agents. In the latter case, the rapid contraction of the choroidal vascular membrane underneath a serous RPE detachment is believed to be the underlying cause. Preservation of good visual acuity after the occurrence of RPE tear with continued use of intravitreal VEGF agents has been reported. In this case report, we describe the occurrence of multiple RPE tears with the use of intravitreal bevacizumab and also correlate the preservation of visual acuity with features seen on spectral domain optical coherence tomography.

Retinal pigment epithelial (RPE) tear is an infrequent complication of pigment epithelial detachment in the elderly, and occurs either spontaneously or after laser photocoagulation.[1,2] Recently, RPE tears have been described after intravitreal bevacizumab injection, due to rapid contraction of the underlying choroidal neovascular membrane (CNVM).[3] The diagnosis is based on typical clinical appearance and fundus fluorescein angiography.[2,3] Retracted RPE after the rip generates increased reflectivity on low-resolution optical coherence tomography (OCT).[4]

Spectral-domain OCT (SD-OCT), a novel imaging technology provides high-resolution cross-sectional images of the retina with high point to point correlation with the fundus picture.[5] We describe SD-OCT features in a patient who developed multiple RPE tears after intravitreal bevacizumab injection.

## Case Report

**Figure 1:**
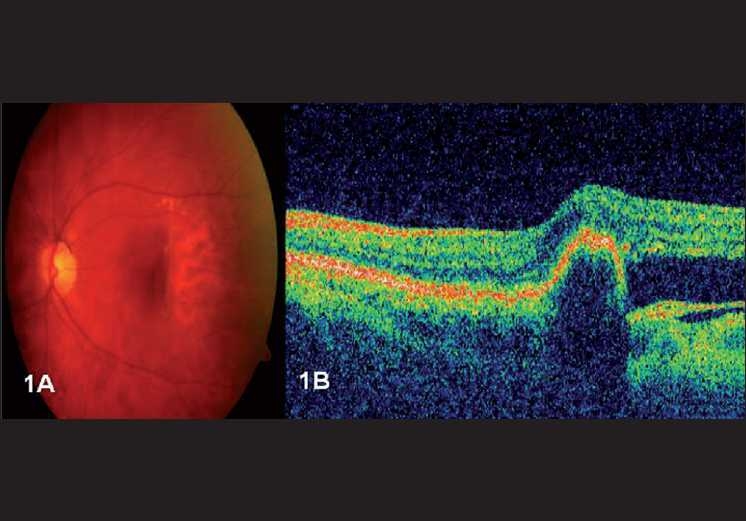
(A) Fundus photograph of the left eye showing the presence of a juxtafoveal retinal pigment epithelial (RPE) tear. (B) Stratus OCT scan of the left eye showing an area devoid of RPE reflectivity with adjoining area of high reflectivity corresponding to the scrolled margin of the RPE tear

**Figure 2:**
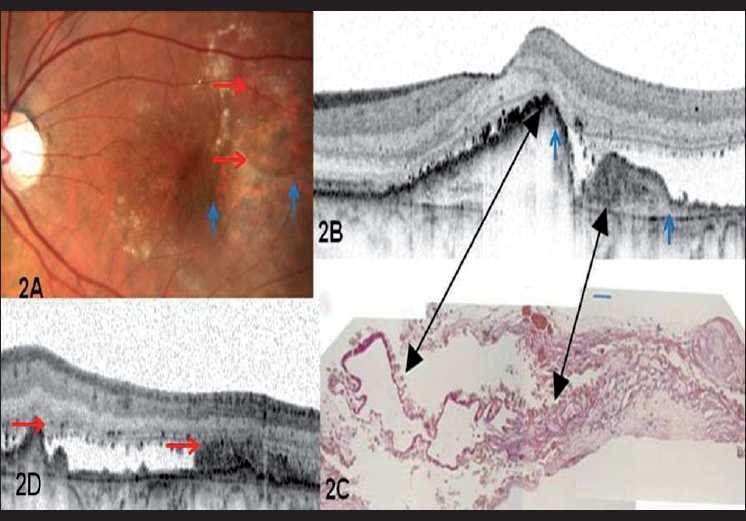
(A) Fundus photograph of the left eye showing the occurrence of a new RPE tear. (B) Spectral-domain OCT of the left eye showing the scrolled up margins of the RPE tear and the fibrovascular membrane lying adjacent to it (area within the blue arrows) (C) Histological picture from a surgical sample showing the corresponding areas of the scrolled up margin of the tear and the fibrovascular membrane (black arrows) (Lafaut *et al*.[6] (D) Spectral-domain OCT of the left eye outlining the second RPE tear (area within the red arrows)

A 70-year-old female with clinical features of advanced exudative age-related macular degeneration presented with bilateral decreased vision. Her best-corrected visual acuities were 20/400 and 20/30 in the right and left eye, respectively. Fundus evaluation revealed a disciform scar involving the macula in the right eye and a serous RPE detachment with an occult CNVM in her left eye for which she received intravitreal bevacizumab, 1.25 mg/ml. One month after the injection, the patient developed a juxtafoveal RPE tear [Fig. 1A]. OCT evaluation with Stratus showed an area devoid of RPE reflectivity corresponding to the tear with an adjoining area of high reflectivity indicating the scrolled up margin of the RPE [Fig. 1B]. Over the next 11 months, four intravitreal injections of bevacizumab were administered to stabilize the CNVM. One year into the follow-up, a new RPE tear was noted, extending from the superior border of the first tear (Fig. 2A, area within blue arrows represents the first RPE tear and the area within the red arrows represents the second tear). Visual acuity in the left eye was maintained at 20/30. SD-OCT (Heidelberg Engineering, Vista, CA / Germany), of the left eye revealed an elevated RPE corresponding to the scrolled margin of the tear [Fig. 2B, 2D]. Fibrovascular membrane beneath the outer segments was seen as hyporeflective, due to the lack of RPE layer. The margin of the rip was subfoveal with elevation of the corresponding area.

## Discussion

The pathogenesis of RPE tear is not completely understood. While some believe that CNVMs play only a minor role in the formation of RPE tears, others consider these membranes to be instrumental in the tearing process.[6,7] Evidence is now in favor of the hydraulic theory, which attributes the development of RPE detachment to the presence of a hydrophobic Bruch’s membrane.[8] It is believed that RPE detachments that are destined to tear tend to become progressively larger and highly detached, generating sufficient tangential stress to cause a rupture. An increase in the incidence of RPE tears is observed after the use of anti-vascular endothelial growth factor (VEGF) agents. This has been attributed to the rapid contraction of the regressing neovascular membrane resulting in tangential traction of the RPE.[3]

The actual composition of RPE tears is not well established. Histological study of the surgically removed RPE tear has revealed that the torn RPE margin rolls up and the bare area of the tear is covered by a thin fibrovascular tissue which covers the remains of the outer segment.[8]

OCT, a useful complementary tool in the clinical assessment of RPE tears reveals high reflectivity signal in the area of the bare choroid and a flattened appearance of the associated pigment epithelial detachment.[4] SD-OCT, also known as Fourier domain OCT collects data 100 times faster than conventional time-domain OCT (TD-OCT), resulting in improved resolution of the B-scan images.[5] The Spectralis, one of the commercially available SD-OCT, also incorporates the Trutrack™technology, which provides reliable point to point correlation between the OCT and the fundus images, thereby allowing repeat scans of identical locations over time. In our patient, SD-OCT showed additional features. The margin of the tear was underneath the subfoveal area with elevation of the corresponding area. The presence of an intact photoreceptor layer overlying the rip margin corresponded to the good visual acuity seen in our patient. In the area devoid of RPE, a raised area with hyporeflectivity was seen corresponding to the fibrous scar.

We used the SD-OCT to define the two features described histopathologically from a surgically excised RPE tear[8] [Fig. 2C]. The scrolled up RPE margin is seen as a hyperintense area with a shadowing underneath. Fibrovascular membrane beneath the outer segments is seen as hyporeflective, due to lack of RPE layer.

The treatment of underlying CNVM after the occurrence of RPE tear is not well established. Continuation of anti-VEGF therapy has been reported to stabilize the visual acuity.[3] In our case, repeated use of bevacizumab may have caused a continued contraction of the fibrovascular membrane with shearing effect resulting in the occurrence of a new tear.

In conclusion, we report formation of multiple RPE tears after repeated use of anti-VEGF therapy for treatment of the underlying CNVM. In addition we correlate the preservation of good visual acuity in the presence of a subfoveal RPE tear with features seen on SD-OCT.
